# L’implication des organisations non gouvernementales dans les systèmes de santé des pays du Sud: l’exemple du Shisong Cardiac Centre

**Published:** 2009-04-27

**Authors:** Jacques Cabral Tantchou Tchoumi, Jean Claude Ambassa, Gianfranco Butera, Alessandro Giamberti

**Affiliations:** 1 St. Elizabeth catholic general hospital Shisong, cardiac unit. Po.Box: 8, Kumbo, Cameroon; 2 Policlinico San Donato, 20097 via Morandi 30, San Donato Milanese

**Keywords:** cardiac surgery, Africa

## Abstract

Dans le tiers monde, près de 800 000 enfants naissent chaque année avec des cardiopathies congénitales (CC). Plusieurs millions d’enfants y vivent avec des CC corrigeables et une grande majorité décède à cause de l’absence de structure médicale spécialisée dans la correction chirurgicale totale ou palliative. C’est dans cette optique que l’association Associazione bambini cardiopatici nel mondo (Association mondiale des enfants cardiopathes) a été créée. Grâce aux efforts de cette association, plus de 500 enfants souffrant de CC ont pu être évacués et soignés à l’hôpital de San Donato et plus 800 dans leur pays. Près de 200 praticiens en provenance des pays du Sud ont dans cette même perspective bénéficiée d’une formation en Italie. Cent quinze missions chirurgicales ont été réalisées dans différents pays: Egypte, Tunisie, Libye, Palestine, Cameroun, Roumanie, Azerbaïdjan, Pérou, Syrie, Yémen, Kurdistan et au Mali. Plusieurs hôpitaux ont été équipés gratuitement. Actuellement, l’association œuvre ardemment pour la réalisation de trois projets essentiels dont l’un est la construction d’un centre cardiaque dans la région de Shisong au Cameroun (Shisong Cardiac Centre). Ce centre cardiaque sera équipé d’appareils de dernière génération permettant de réaliser des investigations et la chirurgie cardiaques. L’inauguration de ce centre est prévue pour Novembre 2009.

## A l’équipe éditoriale du Journal Pan Africain de Médecine

Dans les pays du Sud, près de 800 000 enfants naissent chaque année avec des cardiopathies congénitales (CC). La haute occurrence des maladies cardiaques acquises chez les enfants est attribuable d’une part aux maladies infectieuses comme la fièvre rhumatismale aigue, la maladie de Chagas, la tuberculose, la malnutrition et d’autre part au taux de natalité élevé [1]. Pour ces raisons, l’Organisation Mondiale de la Santé (OMS) a déclaré le traitement et la prévention des maladies cardiovasculaires comme priorité dans un futur très proche [2].

Il faut dire que dans les pays concernés, des millions d’enfants vivent avec des CC corrigeables aussi, une grande majorité décède d’une part à cause du manque de structure médicale spécialisée dans la correction chirurgicale totale ou palliative de ces affections et d’autre part à cause de pauvreté, le coût de la chirurgie cardiaque étant bien élevé. Certes, nous notons l’action des organisations de volontaires et de charité qui offrent une opportunité de prise en charge aux populations défavorisées, mais l’impact de leurs actions est limité. Il peut être amélioré grâce à une meilleure coordination et collaboration des équipes, mais aussi l’implication des journaux et les congrès internationaux dans la diffusion des informations relatifs à la situation des patients avec CC pour une prise de conscience des autorités administratives et politiques pouvant mener à un meilleur financement des projets dédiés à la prévention et au traitement de ces maladies [3]. C’est dans cette lancée qu’en 1994, le chirurgien cardiaque pédiatrique Alessandro Frigiola, de l’hôpital de San Donato à Milan (Italie) et le docteur Silvia Cirri, anesthésiologue à l’hôpital de Sant’Ambroggio Milan fondent l’association dénommée Associazione Bambini Cardiopatici nel Mondo (Association des Enfants avec Cardiopathie du Monde).

Cette association a plusieurs domaines d’intervention; son but principal étant la création de centre autonome de chirurgie cardiaque et leur équipement en appareils modernes afin d’assurer une meilleure prise en charge des patients atteints des CC et maladies cardiaques acquises dans les pays concernés.

L’association réalise ces objectifs à travers des activités de collecte de fonds, de formations pour le personnel médical (infirmiers, techniciens, médecins) en Europe et sur place, des évacuations sanitaires pour les cas de cardiopathie complexes, et la prise en charge locale pour les cas simples. A cela s’ajoute des activités didactiques comme la participation aux congrès nationaux et internationaux et la promotion et le financement de la recherche sur les maladies tropicales [4].

Durant ses 14 ans d’existence, l’association a ainsi permis de soigner plus de 500 patients au centre hospitalier universitaire de San Donato et plus 800 dans les pays concernés; près de 200 médecins originaires des pays du Sud ont obtenus des bourses de formation en Italie. Cent quinze (115) missions chirurgicales ont été réalisées dans différents pays: Egypte, Tunisie, Libye, Palestine, Cameroun, Roumanie, Azerbaïdjan, Pérou, Syrie, Yémen, Kurdistan et Mali. Des équipements médicaux (appareils d’échocardiographies, moniteurs de paramètres, ventilateurs, électrocardiographes, défibrillateurs, bistouris électriques, saturomètres, appareils pour circulation extracorporelle et pompes pour infusion électrique) ont été distribués gratuitement à divers hôpitaux.

Actuellement, l’association œuvre ardemment pour la réalisation de trois projets essentiels: le premier, appelé «Cameroon Project », a démarré en 2001 avec la collaboration des Sœurs Tertiaires Franciscaines propriétaires d’un hôpital général à Shisong et d’un nombre important de centres de santé dans les banlieues de la deuxième ville importante du Nord-Ouest du Cameroun) et une organisation de charité italienne «Cuore Fratello».

Le projet vise au développement du diagnostic et de la correction chirurgicale des maladies congénitales et acquises du cœur au Cameroun. Avec l’aide financière des bienfaiteurs italiens, la construction d’un centre de chirurgie cardiaque dénommé “Shisong Cardiac Centre”, est en cours. Il sera équipé d’appareils modernes pour les investigations et la chirurgie cardiaques. Le centre cardiaque est à proximité de l’hôpital général de Shisong; son inauguration est prévue pour Novembre 2009. Entre 2005 et 2008, 9 missions cardio-chirurgicales ont été réalisées dans cet hôpital, pendant lesquelles 20 malades ont été opérés, plus de 100 patients ont déjà été évacués pour la correction de leur cardiopathie au centre hospitalier universitaire de San Donato (Istituto policlinico San Donato) à Milan et 15 professionnels de la santé ont reçu des bourses de formation [5].

Témoignant de ces actions, la sœur Alphonsa, actuellement vicaire de la congrégation des Sœurs Tertiaires Franciscaines à Rome lors d’une mission chirurgicale déclara : “Le centre cardiaque ne donne pas seulement la vie et la santé aux camerounais, mais aussi un fort sentiment d’appartenir au monde entier”. La force unissante du centre cardiaque devient de plus en plus étonnante prenant en considération la réalité des guerres et les discriminations politiques, sociales et religieuses qui ravagent notre continent. Beaucoup de personnes sont entrées dans notre histoire à travers le don du centre cardiaque et par conséquent, nous sommes appelés à être plus compréhensifs dans la nécessité de collaboration. Vraiment, le centre cardiaque est plus qu’un projet de l’hôpital de San Donato et des Sœurs Tertiaires Franciscaines- c’est une affaire mondiale. Les relations établies dans le processus sont irremplaçables. Nous établissons une différence dans l’histoire du monde et c’est merveilleux de voir, toucher, sentir, et actuellement être part de cela!»

Le second projet est mis en œuvre en Syrie. L’association aide l’université de Damas à construire un département pédiatrique de cardiologie et de cardio-chirurgie. Ce centre est en voie de finition. L’ouverture est prévue dans quelques années.

Le troisième projet est conduit au Pérou. Ce projet est réalisé en coopération avec Essalud, une compagnie nationale d’assurances péruvienne: ayant commencé en 2003 probablement s’achèvera en 2009.

## Conflits d’intérêt

Les auteurs sont tous associes, directement au indirectement au Shisong cardiac center et à Associazione Bambini Cardiopatici nel Mondo
